# Allografts in reconstruction of the posterior cruciate ligament: a health economics perspective

**DOI:** 10.1007/s00167-019-05477-4

**Published:** 2019-03-22

**Authors:** Norman Waugh, Hema Mistry, Andrew Metcalfe, Jill Colquitt, Emma Loveman, Pamela Royle, Nick A. Smith

**Affiliations:** 1 Division of Health Sciences, Warwick Medical School, University of Warwick, Gibbet Hill Campus, Coventry, CV4 7AL UK; 2 Warwick Clinical Trials Unit, University of Warwick, Coventry, UK; 3 Effective Evidence, Waterlooville, Hampshire UK; 4 Department of Orthopaedics, University Hospitals Coventry and Warwickshire, Coventry, UK

**Keywords:** Posterior cruciate ligament, Reconstruction, Cost analysis, Clinical effectiveness

## Abstract

**Purpose:**

To review the relative cost-effectiveness of allografts and autografts in reconstruction of the posterior cruciate ligament.

**Methods:**

Systematic review and cost-effectiveness analysis.

**Results:**

The available evidence does not show any significant difference in clinical effectiveness between autografts and allografts. Given that, only a cost analysis is provided, which shows that allografts are more costly.

**Conclusion:**

Given the lack of any benefit of allografts over autografts, autografts should be preferred on cost grounds, if available. However, there may be situations where an allograft is indicated, for example, in multiple ligament reconstructions.

**Level of evidence:**

IV.

**Electronic supplementary material:**

The online version of this article (10.1007/s00167-019-05477-4) contains supplementary material, which is available to authorized users.

## Introduction

Pache et al. [19] provide a useful overview of the posterior cruciate ligament (PCL), noting that its function is to stop the femur from sliding too far forward on the tibia. Injuries occur during sports such as soccer and rugby, and also in road traffic accidents, when the flexed knee hits the dashboard and the tibia is displaced backwards.

PCL ruptures are much less common than ACL ruptures (about 10% of ACL numbers) and allografts are commonly used, partly because PCL rupture may be part of a multi-ligament problem, when allografts may become necessary due to a lack of graft availability. Such situations do need to be considered when assessing these results, but for the remainder of this report it is assumed that the PCL is an isolated injury, that both autografts and allografts are available, and that a choice can be made between them.

The PCL has much more capacity for healing than the ACL, and many PCL ruptures are treated conservatively, especially if the rupture is partial. Repairs can be a single bundle of the antero-lateral bundle only, or double bundle of both. Pache et al. [19] prefer double-bundle repairs, noting that previous single-bundle repair studies reported that though results were mostly good, many patients were left with posterior laxity which was associated with osteoarthritis (OA) in later years. An earlier systematic review by Chahla et al. [4] used data from 441 patients in 11 studies (mostly not achieving good quality scores, only three were RCTs) to compare the results of double- and single-bundle repairs. Both methods improved knee stability and patient outcomes, but double bundles gave better posterior stability and IKDC scores.

The original aim of the cost-effectiveness analysis was to determine whether an allograft or an autograft is the more cost-effective option for patients requiring a PCL reconstruction. Isolated PCL injuries often respond to conservative care including physiotherapy, but some cases do cause problems despite conservative care and require surgery. The starting point of this analysis was that it had been decided that surgery is required, so conservative care was not included in the economic model.

## Materials and methods

A systematic review was carried out, looking first for good-quality, recent systematic reviews.

Fuller details of methods are given in the full report which will be available at https://www.esska.org, but in brief;

- The databases Ovid Medline, Ovid Embase, Web of Science and the Cochrane Library for articles published from the year 2000 until February 15th 2018. The Medline search strategy and the numbers of records obtained are shown in Appendix 1 of the full report on the ESSKA website.
- Titles and abstracts of retrieved studies were screened by two people, with full papers obtained if inclusion or exclusion was uncertain from the abstract
- Standard systematic review methods were used with quality assessment of included studies using standard checklists for both reviews and primary studies, and checking of data extractions by a second reviewer

For economics analysis, a decision tree model was developed within Microsoft Excel® and was considered the most appropriate choice as reconstruction is usually successful and most patients return to a functioning knee after reconstructive surgery. The starting point for the economic model is at the point where the decision is made to do PCL reconstruction. The clinical pathways were developed using information from the published literature and clinical experience.

However, cost-effectiveness analysis is only worthwhile if there are differences in clinical effectiveness. If there are none reported, one cannot generate utility data and QALYs. A model for analysis was developed but given the paucity of evidence comparing allografts and autografts, and the lack of any significant differences between them in the available studies and analyses, no modelling analyses were undertaken. Therefore, a simple cost analysis is presented comparing the cost of allografts versus autografts for PCL reconstruction. It is assumed that patients have isolated PCL injuries.

### Resource use and costs

All unit costs reported in Table 1 are presented in pounds sterling (£) in 2016/17 prices. The cost of the allograft (£2400) was obtained from the NHS Tissue Services price list for 2018/19 and was based on an average price of a tibialis posterior tendon [16]. There is no cost for the graft in the HS autograft arm, except a little extra theatre time (but the procedure code will not change, so the hospital charge will not vary). Costs include that of the procedure, three consultant-led follow-up clinics, eight physiotherapy sessions and the cost of analgesics (paracetamol and ibuprofen). We have assumed that a second or third PCL revision would cost the same as a first reconstruction.

**Table 1:** Resource use and costs for PCL reconstruction

Resource use	Unit cost (£)	Source
*Graft type*		
Allograft	£2400	Tissue bank [16]
*Procedure*		
Intermediate knee procedures for non-trauma, 19 years and over (HRG code: HN24C)	£1642	NHS reference costs 2015–2016 [9]
*Other related costs*		
Three consultant-led outpatient follow-up attendance (HRG code: WF01A)	£336	NHS reference costs 2015–2016 [9]
Eight hospital physiotherapy sessions (30 min)	£132	UCHSC 2017 [6]
Paracetamol (two tablets twice a day per year)	£23.21	BNF 2016−2017 [3]
Ibuprofen (one tablet a day per year)	£12.47	BNF 2016–2017
*Total costs*		
Allograft	£4395	
HS autograft	£2145	
BPTB autograft	£2145	
*Infection*		
Infections	£7,761	Genuario et al. [8]
*Conservative care*		
One consultant-led outpatient follow-up attendance (HRG code: WF01A)	£112	NHS reference costs 2015–2016 [9]
Eight hospital physiotherapy sessions (30 min)	£132	UCHSC 2016 [7]

It was assumed that 0.3% of all reconstructions will get infections based on a recent ACL study [24]. The cost of infections was obtained from the Genuario et al. [8] paper in US $ in 2010 prices. These costs were converted into UK £ in 2017 prices using the World Bank gross domestic product deflators [25] and the purchasing power parity measures [17]. The cost of treatment for an infection included the cost of requiring debridement, irrigation, and antibiotics started intravenously with 1-week hospital admission, and then continued for a further 5 weeks [2].

## Results

A systematic review by Hudgens et al. [10] included 19 studies, of which 5 were on allografts, 12 on autografts and 2 [1, 23] compared the grafts. Hudgens et al. summarised the advantages of allografts as: shorter operation time, avoidance of donor site morbidity, and a range of graft length and thickness. Disadvantages include deleterious effects on graft strength from sterilisation methods, costs, problems with availability, and a theoretical risk of disease transmission. Some of these hazards are now historical. Disadvantages of autografts included graft size limitations, and the effects of harvesting—increased theatre time, graft site infection, and donor site pain. Hudgens et al. note the scarcity of comparative data but conclude that both grafts give satisfactory results.

Tian et al. [22] provide a meta-analysis of autograft and allografts in PCL reconstruction but include only five studies, the RCT by Li et al. [12], and CCTs by Ahn et al. [1], Li et al. [11], Sun et al. [21] and Wang et al. [23], some of which we would exclude. Ahn et al. [1] had only 18 patients in each group. Tian et al conclude that there is insufficient evidence to say whether autografts or allografts are better.

Systematic reviews that were identified in the searches were used as a source of primary studies. Two studies compared allografts and autografts, Wang et al. [23] and Ahn et al. [1]. Both had weaknesses. Wang et al. [23] randomised patients by day of admission, and lack of availability of allografts resulted in unequal numbers in arms, with 23 allografts (14 Achilles, 9 anterior tibial) and 32 autografts (16 quadriceps, 16 hamstring). The Ahn et al. controlled clinical trial (CCT) compared double loop hamstring autografts used in 1997–1999 with Achilles tendon allografts used in 1999–2000. Six prospective studies of allografts in posterior cruciate ligament (PCL) reconstruction [13–15, 20, 26, 27] were included. Two studies [12, 21] were excluded because grafts were irradiated. There was a range of study designs, with two RCTs of allograft options, one RCT comparing allografts and autografts, two CCTs and four single-arm before and after studies. Study characteristics and baseline characteristics of the participants are summarised in the Supplementary file. The risk of selection bias in the RCTs was unclear, and was high in the CCT. The quality of the single-arm before and after studies was fair in two studies [14, 20] and poor in two studies [15, 27]. The Cooper et al. [5] case series included patients receiving allografts for combined reconstructions and autografts for isolated PCL reconstructions, but 85% of patients had combined reconstructions so in effect it is a case series of only six patients, so of limited value for this analysis. The overall quality and details of each study are reported in Table 2 below and the Supplementary table. Not all are relevant to the primary question.

**Table 2:** Lysholm knee score, PCL reconstruction

Lysholm Knee score at final endpoint, mean (SD) unless stated			*P* value
Ahn [1] unclear ROB	Allograft group *n* = 18	Autograft group *n* = 18	< 0.01
Baseline	68.6	68.2	
Change at endpoint	+ 17.2	+ 21.9	
Li [13] Unclear ROB	Single-bundle allograft, *n* = 22	Double-bundle allograft, *n* = 24	
Baseline value	63.1 (3.8)	64.6 (4.3)	NA
Endpoint value	88.0 (4.2)	89.8 (3.8)	
*P* value	< 0.05^a^	< 0.05^a^	
Wang [23] high ROB	Allograft group, *n* = 23	Autograft, *n* = 32	
Endpoint value	92.3 (6.8)	87.8 (9.6)	n.s
Yoon [26] unclear ROB	Single-bundle allograft, *n* = 25	Double-bundle allograft, *n* = 28	
Median (range)			
Baseline value	64 (41–73)	62 (43–71)	NA
Endpoint value	89 (71–99)	91 (76–100)	
*P* value	< 0.001	< 0.001	
Min [15] poor quality	Allograft, *n* = 21		
Mean (range)			
Baseline value	52.2 (42–66)^b^		
Endpoint value	78 (56–92)^b^		
*P* value	< 0.001		
Lim [14] fair quality	Allograft, *n* = 22		
Median (range)			
Baseline value	64 (50–75)		
Endpoint value	88 (82–96)		
*P* value	< 0.001		
Yoon [27] poor quality	Allograft, *n* = 26		
Baseline value	59.5		
Endpoint value	91.8		
*P* value	< 0.05		

^a^*P* value between baseline and 24 months after surgery, data presented are for final follow-up

^b^Rates different in the abstract, which were 53 (SD 5.3), range 34–68 pre-operatively and 83.5 (SD 13), range 61–97 at follow-up of mean 49.2 months (range 25–73). ROB risk of bias

### Failure and survival

None of the studies reported allograft failure rates or survival of the allograft. Spiridonov et al. [20] reported a rate of implant removal of 7.7% at a mean of 2.5 years follow-up. Li et al. [13] stated that no participants required additional surgery because of recurrent or residual symptoms.

### Functional outcomes

Six studies reported the Lysholm knee score [14, 15, 23, 26, 27]. In the CCT by Wang et al. [23] (high risk of selection bias), there was no statistically significant difference between the allograft group and the autograft group at endpoint (Table 2). All other studies demonstrated a statistically significant improvement in Lysholm score at endpoint. Sample sizes were small and no studies were of low risk of bias/good quality; Lim et al. [14] was of fair quality.

Four studies [13, 14, 23, 26] reported the Tegner score (Table 3). There was no statistically significant difference between allografts and autografts in the Wang et al. study. All other studies found a statistically significant improvement in Tegner score at follow-up, although no studies were of low risk of bias/good quality and sample sizes were small.

**Table 3:** Tegner scores, PCL reconstruction

Tegner score at final endpoint, mean (SD) unless stated			*P* value
Li [13] unclear ROB	Single-bundle allograft, *n* = 22	Double-bundle allograft, *n* = 24	
Baseline value	3.1 (0.6)	3.3 (1.0)	NA
Endpoint value	6.2 (0.9)	6.8 (1.2)	
*P* value	< 0.05^a^	< 0.05^a^	
Wang [23] high ROB	Allograft group, *n* = 23	Autograft, *n* = 32	
Endpoint value	4.70 (1.66)	4.73 (1.66)	n.s
Yoon [26] unclear ROB	Single-bundle allograft, *n* = 25	Double-bundle allograft, *n* = 28	
Median (range)			
Baseline value	2 (1–3)	2 (1–3)	NA
Endpoint value	6 (4–7)	6 (4–7)	
*P* value	< 0.001	< 0.001	
Lim [14] fair quality	Allograft, *n* = 22		
Median (range)			
Baseline value	3 (2–5)		
Endpoint value	6 (3–9)^b^		
*P* value	< 0.01		

^a^*P* value between baseline and 24 months after surgery, data presented are for final follow-up

^b^States mean, reviewer assumed this is median as per the other outcomes

Four studies assessed the IKDC subjective score as shown in Table 4. In the Yoon et al. [26] and Spiridonov et al. [19] studies that compared endpoint results with baseline, for allografts only, statistically significant improvements were seen. Cooper et al. [13] found similar results with autografts and allografts.

**Table 4:** IKDC subjective, PCL reconstruction

IKDC subjective at final endpoint, mean (SD) unless stated			*P* value
Ahn [1]	Autograft *n* = 18	Allograft *n* = 18	
Final follow-up	16 normal, 2 abnormal	14 Normal, 4 abnormal	n.s
Li [13] unclear ROB	Single-bundle allograft, *n* = 22	Double-bundle allograft, *n* = 24	
Endpoint value	65.5 (7.8)	71.6 (6.7)	NA
Yoon [26] unclear ROB	Single-bundle allograft, *n* = 25	Double-bundle allograft, *n* = 28	
Median (range)			
Baseline value	40.2 (27.6–46.0)	39.1 (27.6–48.3)	NA
Endpoint value	79.3 (59.8–88.5)	81.7 (65.5–88.5)	
*P* value	< 0.001	< 0.001	
Spiridonov [20] fair quality	Allograft, *n* = 39		
Baseline value	39.3 (18.8)		
Endpoint value	74.3 (23.1), *n* = 31		
*P* value	< 0.0001		
Cooper [5]	Allografts 25, autografts 16, but only 6 autografts in isolated PCLR		
Baselines all severely abnormal based on stability	Average final score 75 (20–100) with slightly better scores with BPTB autografts than BPTB allografts (76 versus 74, *P* < 0.05, but clinically not significant), but overall results are similar. But different graft thicknesses and mixture acute and chronic		

### Quality of life

No studies of PCL reported quality of life.

### Adverse events

Of the seven studies, only three reported any specific complications or adverse events and rates were generally low. Yoon et al. [26] reported post-operative limited range of motion in 4% and 7% of participants in the single-bundle allograft and double-bundle allograft groups, respectively. Wang et al. [13] reported one complication (organism isolated from wound but no clinical infection) in the allograft group and seven (21.9%; two infections, four donor site pain, one reflex sympathetic dystrophy) in their autograft group. Min et al. [13] reported arthrofibrosis in one case and irritation leading to tibial screw removal in four of their 21 allograft cases. Li et al. [13] reported no major neurologic, vascular, or wound complications. Spiridonov et al. [20] reported no intra-operative neurovascular injuries, deep vein thrombosis or infections, and Lim et al. [14] reported no complications. Yoon et al. [27] did not report adverse events.

### Cost analysis

Table 5 shows the base-case discounted cost results. Having an allograft as a primary ACL reconstruction is more costly (£2455 more) than having a HS autograft as a primary ACL reconstruction. The main cost driver for this result was the cost of the graft.

**Table 5:** Base-case discounted results, PCL reconstruction

Procedure	Total mean costs £	Incremental costs
HS autograft	£2426	–
Allograft	£4881	£2455

## Discussion

Most studies cited in this review were observational studies, with only one relevant RCT, and two CCTs, most with only about 20 or so patients, and none being rated as having a low risk of bias. The RCT by Wang et al. [23] was rated at high risk of bias, partly because of the method of randomisation (according to day of admission), and partly because of problems with the supply of allografts, which were not always available. Some older trials were excluded because allografts were irradiated, sometimes with 2.5 Mrad, sometimes with an unspecified dose. Most studies were of allografts only, sometimes comparing single bundle with double bundles. Clinical practice in our centre is that if a double-bundle PCL reconstruction is being done, allografts are more likely to be used, the view being that double-bundle PCL reconstruction would require two ligament autografts. However, from the comparative studies in this review, it appears that short-term results from small studies are similar for single bundle and double bundles [13, 26]. The evidence of reduced posterior laxity in double-bundle reconstructions raises the possibility of a difference in long-term outcomes appearing over time but that is theoretical only and has no current evidence base, and so is not considered in this analysis.

Ahn et al. [1] summarised the advantages of using Achilles tendon allografts as decreased surgical time, absence of donor site morbidity, and bone to bone fixation, and the disadvantages are cost, longer time to heal, and possibly tendency to stretch. They considered the disadvantages of double-loop hamstring tendon autografts to be short length (10–11.5 cm) and harvesting time, and morbidity.

This analysis started from the decision to operate and surgical reconstruction was not compared with non-operative care. A study by Oweson et al. [18] examined the cost-effectiveness of surgical reconstruction for isolated PCL injuries, looking at both single-bundle autograft and double-bundle allograft, compared to non-surgical care (dynamic brace, 6 months of physiotherapy rehabilitation, with 2 sessions a week, and independent training). They expressed cost-effectiveness in terms of cost per KOOS point. They considered that single-bundle autograft reconstruction was cost-effective compared to non-surgical care. Of interest to this review, they also compared double-bundle allograft to single-bundle autograft, noting the extra cost (allograft 4200 euros plus some fixation costs) but failed to find convincing evidence that double bundle was sufficiently more effective to justify the extra costs.

In practice around half of PCL ruptures are part of multi-ligament injuries where graft availability limits the choices of the surgeon, and the findings of this review may not apply.

## Conclusion

The before and after evidence shows benefit from the use of allografts, but does not provide any evidence for their superiority over autografts in isolated reconstruction of the PCL. Given the extra cost of allografts, they do not seem justified if autografts are available, and if there is no clinical reason to prefer an allograft. However, PCL injuries often occur as part of multi-ligament knee injuries, where availability of suitable autografts may be an issue.

## Electronic supplementary material

Below is the link to the electronic supplementary material.

Supplementary Material 1 (DOCX 41 kb)

## Abbreviations

PCL: Posterior cruciate ligament
OA: Osteoarthritis
ACL: Anterior cruciate ligament
IKDC: International knee documentation committee
RCTs: Randomised controlled trials
CCT: Controlled clinical trial
