# Supplementary material for: Allografts in reconstruction of the posterior cruciate ligament: a health economics perspective
Source: Knee Surg Sports Traumatol Arthrosc. 2019 Mar 22;27(6):1810–6. doi: 10.1007/s00167-019-05477-4 (PMC6541577; doi:10.1007/s00167-019-05477-4)
Supplement: Supplementary file 1 — Supplementary Material 1 (DOCX 41 kb) [file 167_2019_5477_MOESM1_ESM.docx]

# **KSST-D-18-01635.** Waugh et al. Allografts in reconstruction of the posterior cruciate ligament: a health economics perspective

## Supplementary Table: Studies and baseline characteristics: PCL reconstructions

| **Study** | **Indication / inclusion criteria** | **Concomitant procedures** | **Baselines** |
| --- | --- | --- | --- |
|  |  |  |  |
| **Ahn et al[1]**  **Country:** Korea  **Study design**. CCT  **Follow-up duration**: minimum 2 years, mean FU 35 months for autografts, 27 months for allografts.  **Sample size:** 36 | Isolated PCL injuries and instability I patients with remnant PCL fibres. Patients requiring multiple reconstructions excluded. From 1997to 1999, double loop hamstring autografts used. From 1990 t0 2000, Achilles allografts used. | Autograft group: three had meniscal surgery.  Allograft group: four partial meniscectomies. | **Age:** upper limit 60 years. Mean ages 30 (range 16 -58) autografts and 31 (range 17 to 60) allografts.  % male: 83% autografts; 67% allografts |
| **Cooper et al[2]**  **Country:** USA  **Study design: case series**  **Follow-up:** minimum 2 years, mean 39 months  **Sample size:** 44 | Isolated or combined PCL reconstructions. 85% were combined.  35 primary reconstructions and 6 revisions  Only 14% (6 patients) had isolated PCL reconstructions, all with autografts. All combined reconstructions used allografts | ACL and MCL repairs in most. Only 6 patients had autografts for isolated PCLR. Limited use for our purposes | Age not given**.**  70% male |
| **Li et al, 2014[3]**  **Country:** China  **Study Design**: RCT (comparison not relevant to review)  **Follow-up duration**: SB 28.7 months (SD 3.0); DB 30.4 months (SD 5.1)  **Sample size:** 50 (SB 25, DB 25) | Isolated posterior knee instability grade II to III, 2007 – 2009. Two groups double-bundle (DB) technique and single-bundle (SB) technique compared | Not reported | **Age^a^ :** SB 25.1 (SD 2.6); DB 23.5 (SD 5.2)  **% male:** SB 68.2; DB 75.0 |
| **Yoon et al, 2011[8]**  **Country:** Korea  **Study Design**: RCT (comparison not relevant to review)  **Follow-up duration**: SB 31 months (range 24-42); DB 33 months (range 24-43)  **Sample size**: 60 (SB 30, DB 30) | Arthroscopic PCL reconstruction for an isolated PCL tear, 2005-2007. Two groups double-bundle (DB) technique and single-bundle (SB) technique compared | Not reported | **Age^a^ :** SB 28.5 (17-47); DB 27.4 (18-46)  **% male:** SB 80.0; DB 89.3 |
| **Wang et al, 2004[7]**  **Country:** Taiwan  **Study Design**: CCT  **Follow-up duration**: mean 34 months (SD 10, range 24-71)  **Sample size:** 55 (23 allograft, 32 autograft) | Indications included pain and instability as a result of high-energy posterior cruciate ligament injury with failure of conservative treatments for 3 months. 1997-2001. | 50% had meniscectomies, meniscus repairs, and/or debridement | **Age^a^ :** Allograft 30 (SD 12); autograft 29 (12)  **% male:** Allograft 69.6; autograft 78.1 |
| **Min et al, 2011[5]**  **Country:** Korea  **Study design:** Before and after study (authors’ definition case series)  **Follow-up duration**: 51.7 months (range, 25-73 months).  **Sample size:** 21 | Indication was painful instability above daily activities in active patients and a PCL injury with >10 mm side-to-side difference. All had isolated PCL rupture with or without meniscal injury and grade III posterior instability. 2003-2007. | Partial meniscectomy in 19%. | **Age^a^ :** 35.6 (18-54)  **% male:** 90.5 |
| **Spiridonov et al, 2011[6]**  **Country:** USA  **Study design:** before and after study  **Follow-up duration**: 2.5 (2.0 – 4.3) years  **Sample size**: 39 | Evidence of an unstable knee with acute multiple ligament injuries, a chronic PCL tear that had not responded to non-operative treatment, or a chronic combined injury of the PCL and posterolateral or medial and/or posteromedial ligaments of the knee were enrolled. Indication grade-III isolated or combined PCL tears. 2005 – 2008. | 10.3% had initial proximal tibial biplanar osteotomy. 82% had combined procedures (PCL with range of medial knee reconstruction; PLC reconstruction, ACL).  . | **Age^a^ :** 33 (15 – 62)  **% male:** 84.6 |
| **Lim et al, 2010[4]**  **Country:** Korea  **Study design:** Before and after study  **Follow-up duration**: 33 (24-60) months  **Sample size**: 22 | Pain or instability during daily activities despite non-operative treatment for more than 6 months, and a PCL injury with more than an 8 mm side-to-side difference in posterior displacement. | Not reported | **Age^a^ :** 36 (18-59)  **% male:** 86.4 |
| **Yoon et al, 2005[9]**  **Country:** Korea  **Study design^a^:** before-after study (author’s description case series)  **Follow-up duration**: 25 months (range 12-48)  **Sample size:** 26 (27 knees) | Underwent arthroscopic double-bundle PCL augmentation using split Achilles allograft 1999-2002. All had contact mechanisms of injury. | 35% with combined ACL deficiency had arthroscopic reconstruction using tibialis anterior allograft, 57.7% meniscectomy or meniscorrhaphy. | **Age^a^ :** 27.9 (17-43)  **% male:** 73.1 |

^a^mean (range) unless stated otherwise.

**References**

1. Ahn JH, Yoo JC, Wang JH (2005) Posterior cruciate ligament reconstruction: double-loop hamstring tendon autograft versus Achilles tendon allograft--clinical results of a minimum 2-year follow-up. Arthroscopy 21:965-969

2. Cooper DE, Stewart D (2004) Posterior cruciate ligament reconstruction using single-bundle patella tendon graft with tibial inlay fixation - 2-to 10-year follow-up. Am J Sports Med 32:346-360

3. Li Y, Li J, Wang J, Gao S, Zhang Y (2014) Comparison of single-bundle and double-bundle isolated posterior cruciate ligament reconstruction with allograft: a prospective, randomized study. Arthroscopy 30:695-700

4. Lim HC, Bae JH, Wang JH, Yang JH, Seok CW, Kim HJ, et al. (2010) Double-bundle PCL reconstruction using tibial double cross-pin fixation. Knee Surg Sports Traumatol Arthrosc 18:117-122

5. Min BH, Lee YS, Lee YS, Jin CZ, Son KH (2011) Evaluation of transtibial double-bundle posterior cruciate ligament reconstruction using a single-sling method with a tibialis anterior allograft. Am J Sports Med 39:374-379

6. Spiridonov SI, Slinkard NJ, LaPrade RF (2011) Isolated and combined grade-III posterior cruciate ligament tears treated with double-bundle reconstruction with use of endoscopically placed femoral tunnels and grafts: operative technique and clinical outcomes. J Bone Joint Surg Am 93:1773-1780

7. Wang CJ, Chan YS, Weng LH, Yuan LJ, Chen HS (2004) Comparison of autogenous and allogenous posterior cruciate ligament reconstructions of the knee. Injury 35:1279-1285

8. Yoon KH, Bae DK, Song SJ, Cho HJ, Lee JH (2011) A prospective randomized study comparing arthroscopic single-bundle and double-bundle posterior cruciate ligament reconstructions preserving remnant fibers. Am J Sports Med 39:474-480

9. Yoon KH, Bae DK, Song SJ, Lim CT (2005) Arthroscopic double-bundle augmentation of posterior cruciate ligament using split Achilles allograft. Arthroscopy 21:1436-1442
